# Uncovering packaging features of co-regulated modules based on human protein interaction and transcriptional regulatory networks

**DOI:** 10.1186/1471-2105-11-392

**Published:** 2010-07-22

**Authors:** Lina Chen, Hong Wang, Liangcai Zhang, Wan Li, Qian Wang, Yukui Shang, Yuehan He, Weiming He, Xu Li, Jingxie Tai, Xia Li

**Affiliations:** 1 College of Bioinformatics Science and Technology, Harbin Medical University, Harbin, Hei Longjiang Province, China; 2 Institute of Opto-electronics, Harbin Institute of Technology, Harbin, Hei Longjiang Province, China

## Abstract

**Background:**

Network co-regulated modules are believed to have the functionality of packaging multiple biological entities, and can thus be assumed to coordinate many biological functions in their network neighbouring regions.

**Results:**

Here, we weighted edges of a human protein interaction network and a transcriptional regulatory network to construct an integrated network, and introduce a probabilistic model and a bipartite graph framework to exploit human co-regulated modules and uncover their specific features in packaging different biological entities (genes, protein complexes or metabolic pathways). Finally, we identified 96 human co-regulated modules based on this method, and evaluate its effectiveness by comparing it with four other methods.

**Conclusions:**

Dysfunctions in co-regulated interactions often occur in the development of cancer. Therefore, we focussed on an example co-regulated module and found that it could integrate a number of cancer-related genes. This was extended to causal dysfunctions of some complexes maintained by several physically interacting proteins, thus coordinating several metabolic pathways that directly underlie cancer.

## Background

One of key challenges of the post-genomic era is to understand the complexity of molecular networks, and describe their applications to elucidate essential principles of cellular systems and disease machinery [1,2]. Spurred by advances in technology, several types of molecular networks, *e.g*. protein-protein interaction networks (PPINs), transcriptional regulatory networks (TRNs), and phenotype networks have been identified, providing us with a global landscape of how biological molecules may interact with one another. Many studies have demonstrated that PPINs and TRNs are essential for controlling the expression levels of genes and the activity of proteins, which mediates coordinated responses and adapted modifications to multifarious cellular stimuli [3,4]. Given this landscape, integrative analysis of both PPINs and TRNs is a major focus in systems biology and bioinformatics. Many computational strategies based on integrated PPIN and TRN networks have been devised and used to decipher specific network structures [4,5] or their potential biological implications [6] that underlie disease traits.

In molecular networks, genes, proteins, and other molecules form components called 'functional modules' that are densely interconnected, but relatively isolated from other networks [7]. Recent surveys have shown that genes within a module or a cluster appear to have similar expression patterns, share common underlying regulatory mechanisms, and thus have strong associations with specific biological functions that determine the behaviour or phenotype of the cell [8,9]. Complex diseases are known to result from the loss of one or more normal essential functions. One such example is cancer. In the recent years, an increasing number of cancer studies have combined human gene expression profiling and computational-based module searching algorithms to obtain a more comprehensive view of the molecular underpinnings and regulatory relationships of cancer [10]. Segal *et al*. [11] have identified gene sets with similar behaviour across microarrays, and constructed 'cancer module maps' to characterize a variety of clinical conditions. Whitfield *et al*. [12] have detected modules in which genes shared both similar expression profiles and similar transcription factor binding profiles. Pomeroy *et al*. [13] have explored regulatory modules using the conservation of co-expression relationships across a diverse range of organisms. The utility of microarray analysis provides more interpretable results than using gene lists alone. A study by Chuang *et al*. [14] have combined microarray analysis and the human PPIN to identify sub-network biomarkers for breast cancer, and proposed that integrated network-based approaches could help researchers acquire additional and more accurate molecular mechanisms for cancers. Another study by Cui *et al*. [15] have demonstrated that the co-regulatory mechanism of molecular networks could mediate cancer-related genes, convey their abnormal states through several functional modules, and eventually lead to uncontrolled cell growth, invasion, and metastasis in distant planes of the body. Thus, uncovering co-regulated modular structures in integrated molecular networks could provide valuable insights into the pathogenesis of cancer.

In this paper, we introduce a probabilistic model termed Co-Regulatory Analysis using Integrated Networks (CRAIN) to detect human co-regulated modules using an integrative weighted network of a PPIN and a TRN. Then the performance of our analysis is evaluated by cross-validation with biological evidence. Furthermore, we figure out biological relevance of our modules for assembling or rewiring biological entities such as genes, protein complexes, and metabolic pathways. Finally, exemplified by cancer, we investigate whether co-regulated modules are capable of assembling different biological entities with underlying mechanisms in tumorigenesis.

## Results and Discussion

### Overview of the identification of co-regulated modules

We scaled and merged a human PPIN and TRN, and constructed a highly quality integrated network of protein and transcription regulation interactions. Adopting a probabilistic model, we evaluated whether a cluster of co-regulated proteins was likely to form a module in the integrated network. Under this model, we formulated a log-likelihood ratio to compare the fit of a cluster to the desired structure with its likelihood, given that the interaction map was randomly constructed. Highly scoring sub-networks corresponded to likely modules. We used a heuristic strategy for module-detecting procedures consisting of: *(i) *seed initialization; *(ii) *seed expanding; and *(iii) *overlap filtering. Finally, we obtained 96 co-regulated modules (Additional file 1), each of which was co-regulated by one or more specific transcription factors (TFs). And furthermore, we used three bipartite graphs to map our modules onto the biological entities of genes, protein complexes, and metabolic pathways to uncover the underlying biological significance of the modules. From our analysis, we concluded that in each module, co-regulated relationships might play important roles in packaging their binding genes, then extending to regulating complexes maintained by several physical interacting proteins, and thus involving in some metabolic pathways or disease traits.

### Performance evaluation

#### Analysis of module robustness

**Figure 1:**
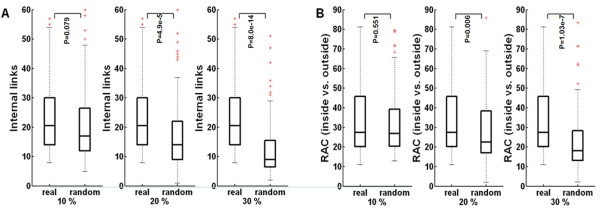
**Robustness analysis of 96 co-regulated modules**. (A) Internal connectivity of detected modules (real) and their controls (random). (B) The ratio of average connections (RAC) (inside vs. outside). P: the significant level of Wilcoxon's rank-sum test.

We assessed the internal connectivity of each co-regulated module by comparison with its control clusters. To generate a control for a given module, we conducted random replacements for 10%, 20% or 30% of the module nodes with an equal number of proteins/TFs outside the module. We repeated this replacement process 100 times, and used the average connectivity for all analytical runs. Figure 1A shows the internal connectivity of the extracted modules and their controls. Inside connections of co-regulated modules decreased significantly with an increase in the replacement size during randomization experiments. We also studied the average connecting ratio of the nodes within each module to the ones outside of it. We found that the ratio in the real dataset was higher than in the randomization experiments (Figure 1B), suggesting that each of the identified modules was indeed densely connected, and robustly formed a local sub-network.

#### Analysis of module functional coherency

Using the TANGO toolkit [16], we performed Gene Ontology (GO) enrichment analysis for our extracted 96 modules, to identify strongly-associated functional categories. The TANGO algorithm includes all levels of GO, and computes raw enrichment p-values using a standard hyper-geometric test with a significant level of p < 0.001. Annotation results showed that 77 modules (80%) were significantly enriched in biological function (Additional file 2).

**Figure 2:**
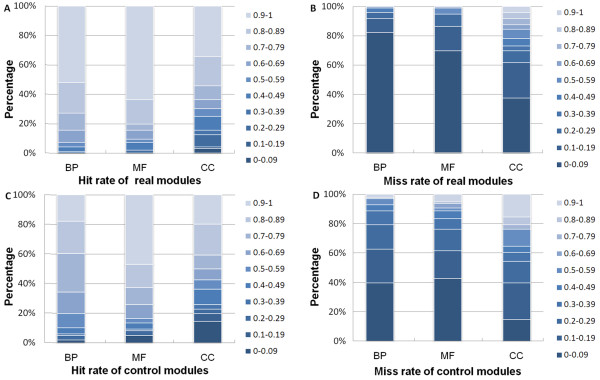
**Functional consistency analysis of 96 co-regulated modules vs. random control modules (30% nodes replacement)**. Horizontal axis symbols represent three branches of GO (BP: biological process, MF: molecular function, CC: cellular component). Vertical axis symbols represent the corresponding percentages with different Hit-rates or Miss-rates for all modules.

To quantify the functional consistency of each discovered module, we computed the Hit-rate and Miss-rate proposed by Milenkoviae *et al*. [17] for each module *M *(GO enrichment significant level p < 0.001):

For a given module *M*,  ( *i = 1, 2,..., t*, where t represents the number of GO terms for which the module *M *enriched) is the intersection gene set of module *M *and its enriched GO term *i*, and |*M*| is the size of *M*. A higher Hit-rate indicated that more genes in module *M *convey a centralized biological function; a lower Miss-rate provided additional confirmation of our deduction. We binned the Hit-rates and Miss-rates in grades of 10%, and compared the Hit-rates and Miss-rates between our predicted modules and their controls (30% nodes replacement) (Figure 2). In the GO: biological process (BP) branch, 50 investigated modules in the real team had a Hit-rate above 90%, and 79 had a Miss-rate below 10%, while 17 modules in the control team had a Hit-rate above 90%, and 38 had a Miss-rate below 10%. The same observations for higher Hit-rate and lower Miss-rate were seen when analyzing the functional consistency of our investigated modules in the molecular function (MF) and cellular component (CC) categories. These results suggested that our method was capable of finding co-regulated modules with strong biological relevance. Similar results were found for the 10% and 20% node replacements (data not shown).

#### Multiple methods comparison

**Figure 3:**
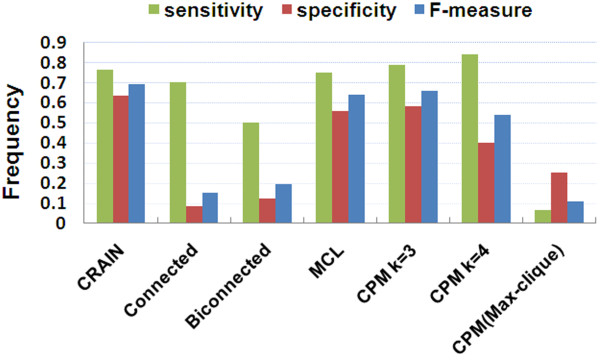
**Performance information of CRAIN, Connected, Biconnected, MCL and CPM**.

We validated the performance of CRAIN by comparison with four other module identification algorithms [18-20]: connected components (Connected), biconnected components (Biconnected), clique percolation method (CPM), and Markov cluster algorithm (MCL). For this process, we predicted modules using these four methods. Enrichment was computed using the standard hyper-geometric test by TANGO toolkit (significance level p < 0.001). For each method, we defined sensitivity as the proportion of annotations enriched in at least one module at p < 10^-4^, and specificity as the proportion of modules enriched with at least one annotation at p < 10^-4 ^[21]:

The F-score summarizes the two measures, and is defined as follows:

Figure 3 is a histogram of three measures: sensitivity, specificity and the summary measurement F-measure, for each algorithm. The results indicated that the F-score of our method was superior to the other methods. This suggested that CRAIN could return co-regulated modules with more affluent biological meanings.

### Biological association of co-regulated modules with cancer

Cancer-related genes are often assumed to mediate each other through the co-regulatory mechanisms of molecular networks, causing abnormal states through several functional modules, and eventually leading to uncontrolled cell growth, invasion, and metastasis to distant planes of the body [15]. To investigate this, we used Fisher's exact test to check biological associations between cancer-mutated genes and each of the 96 identified modules. We found that 42 (43%) of modules were associated with cancer (p < 0.05, Additional file 3).

### Packaging features of co-regulated modules

Furthermore, to determine the biological importance of the co-regulated modules, we investigated the role of transcription regulation in assembling or rewiring genes, protein complexes, and metabolic pathways within modules.

#### Gene packaging

**Figure 4:**
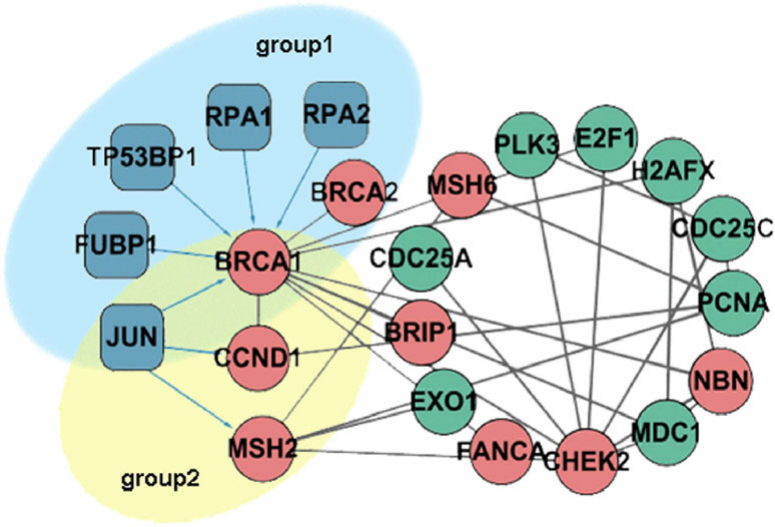
**A sample module associated with 'biopolymer metabolic process'**. Blue nodes represent TFs, red nodes represent cancer-mutated genes, and green nodes represent non-mutated genes. Interactions between TFs and proteins are blue; interactions between protein pairs are gray.

For all 96 co-regulated modules, we labelled TFs and proteins with their associated biological functions. We found that each module could work as an 'assembler' to assemble a set of genes with similar biological functions that were regulated by one or more TFs. For example, Figure 4 illustrates one module associated with a 'biopolymer metabolic process' (module 27). In this module, two groups of regulated subsets were identified: one group consisted of *JUN *and three tumour-mutated genes (*CCND1*, *MSH2 *and *BRCA1*). Recent studies have reported that *JUN*, a key cancer-related regulator, is important in carcinogenesis: inappropriate gene activation or numerous different genetic defects of *JUN *or its target genes could lead to cell growth inhibition, DNA damage or cell cycle delay, and these series of unexpected variations could finally have effects on tumour emergence, promotion and metastasis [22,23]. Another group contained five TFs (*RPA1*, *RPA2*, *TP53BP1*, *FUBP1*, and *JUN*) and their target genes (*BRCA1 *and *BRCA2*). *BRCA1 *and *BRCA2 *are important tumour suppressor genes, whose loss of function is closely associated with tumorigenesis [24,25]. Several studies have reported that these two genes are involved in DNA recombination and DNA repair [26-28]. A mutation in *BRCA1 *or *BRCA2 *compromises interaction with replication protein A (*RPA1 *and *RPA2*), and these two proteins are essential for DNA replication, repair, and recombination [29,30]. Lack of interaction first inhibits the recruitment of double-strand break repair proteins, then leads to an accumulation of carcinogenic DNA abnormalities, eventually causing predisposition to early onset cancer. These findings demonstrated that one or more TFs in co-regulated modules could package different genes with specific functions. Cancer-related modules could assemble a set of cancer-mutated genes and regulate specific biological functions associated with cancer, thus contributing to the pathogenesis of disease traits.

To address whether genes that link to genes mutated in cancer in co-regulated modules are more likely to be cancer-associated, we interrogated non-mutated genes within modules associated with 'biopolymer metabolic process' (module 27), using manual literature validation. We found that all non-mutated genes were implicated in tumorigenesis (Additional file 4). These results suggested that genes in cancer-related co-regulated modules had a high disease risk for tumours, and might be tumour candidate biomarkers. Additional analysis found that similar results could be obtained for all other cancer related co-regulated modules (data not shown).

#### Complex packaging

**Figure 5:**
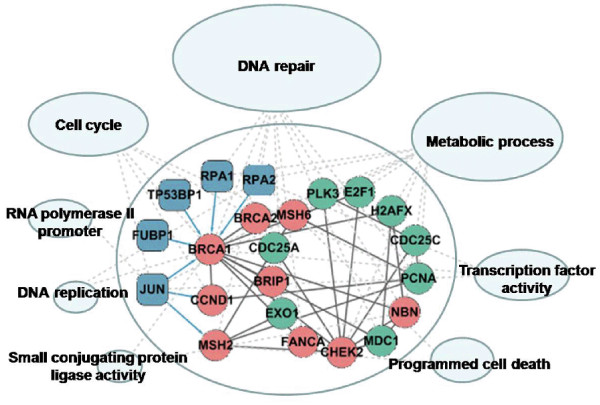
**Complex packaging of the 'biopolymer metabolic process' module**. As an assembler, this co-regulated module organized eight groups of protein complexes. The ellipses mark groups of complexes belonging to similar functional classes. Node size depends on the number of complexes in each function group.

To access the association of co-regulated modules with protein complexes, we acquired 1347 human protein complexes from the MIPS database as a reference set, and analyzed the packaging characteristic of our modules [31,32]. A hyper-geometric test was used to evaluate the significance of overlap between our modules and the MIPS functional categories. The results showed 90 (94%) modules that could organize numerous protein complexes (p < 0.05, Additional file 5). As an example of these significant results, a sample module that is involved in 'biopolymer metabolic process' (module 27), packages 98 protein complexes involved in eight functional classes (Figure 5, Additional file 6). The complexes and this module share a set of cancer-related functions such as DNA repair, cell cycle regulation, and transcription from RNA polymerase II promoters. Many studies have shown that gene alterations in cancer patients, such as malignant changes in DNA sequence and chromosomal fragment amplifications, cause subtle divergence of the DNA sequence with subsequent mistakes in replication during 'DNA repair' and 'DNA replication', altering 'transcription activity' and 'cell cycle', resulting in the evolution of mutinous cells, and resulting in the ability to invade and metastasise [33-37]. Similar packaging results for the other co-regulated modules are in additional file 5. These results suggested that our co-regulated modules had the functionality of rewiring different protein complexes, and that cancer-related modules could package complexes that underlie carcinogenesis.

#### Pathway packaging

**Figure 6:**
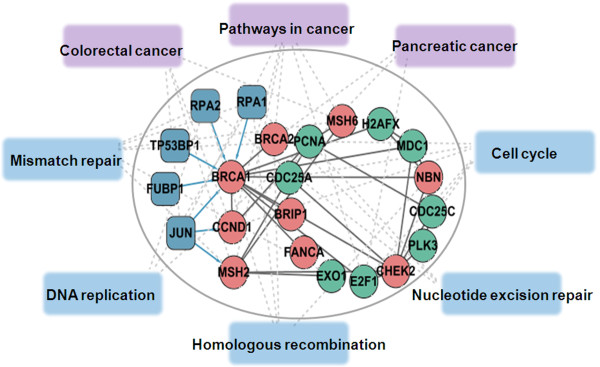
**Pathway packaging of the 'biopolymer metabolic process' module**. As an assembler, this co-regulated module organized eight divergent metabolic pathways. Cancer pathways are in purple, and cancer-related pathways are in blue.

To further investigate the assembling power of co-regulated modules on metabolic pathways, we performed KEGG annotation analysis for each module using DAVID (Count > = 2; EASE < = 0.05) [38,39], a useful tool that integrates different sources of biological information to obtain biological annotations, and ranks them by statistical significance. We found that 79 (82%) modules had significant annotated pathway information (Additional file 7). A sample module ('biopolymer metabolic process') assembled eight divergent metabolic pathways (Figure 6). We discovered two cancer-related TFs (*RPA1 *and *RPA2*) that function as hub TFs, forming focal nodes in information exchange between eight metabolic pathways. These two TFs and their binding proteins in the module work in a complementary manner to rewire the mismatch repair, cell cycle, and homologous recombination pathways leading to the dysfunction of different cancer pathways [40-42]. In our prior studies, we found that genes in cancer development and progression are distributed sparsely among different metabolic pathways. According to pathway analysis, we concluded that our modules had the functionality of organizing multiple biological pathways and controlling numerous cell behaviours, which eventually contribute to cancer pathogenesis.

## Conclusions

We devised and implemented a probabilistic model and a bipartite graph framework to infer human co-regulated modules. We analyzed their specific features in packaging different biological entities from an integrated molecular network with high confidence. Through robustness analysis, we demonstrated that our algorithm identified probable co-regulated modules for *Homo sapiens*. The performance of our approach was evaluated by comparison with other four module identification approaches. Further analysis using the bipartite graph framework uncovered packaging features for co-regulated modules, and showed that modules appeared to act as 'assemblers' dominated by several transcriptional regulations, and tended to coordinate complexes maintained by several physical interacting proteins, and indicating involvement in metabolic pathway cross-talk within neighbouring regions.

The success of our method can be attributed to the following factors. PPINs and TRNs are based on the curated literature and experimentally-determined interactions, so an integrated molecular network can be used to identify co-regulatory modules. In addition, we introduced a bipartite graph framework to evaluate packaging features of co-regulated modules with different biological entities, which easily divided biological entities into piles according to each module. As shown by various examples, our method appears to be effective in the identification of human co-regulated modules, and in searching for their packaging features in biological entities.

However, our proposed method has some limitations. We introduced a greedy algorithm aimed to make the locally optimal choice at each expanding step. Greedy algorithms are known to generally fail in finding globally optimal solutions, because they usually do not operate exhaustively on all the data. However, from our analysis results, we believe that the greedy algorithm was effective for module identification. The limitations of the proposed method for packaging (overlap) analysis are that two-thirds of human genes are annotated by at least one functional annotation, but the remaining one-third has yet to be annotated [43]. In addition, the incompleteness of information about complexes and biological pathways might miss some significant overlaps or packing relationships. Although our proposed method has these limitations, the packaging features of co-regulated modules could still be deciphered in integrated molecular networks. With the accumulation of human data, we expect that our framework may facilitate the identification of additional modules and their packaging features.

## Methods

### Human interaction data sources

Human protein-protein interaction data was extracted from the HPRD databases (Release7) [44]. The derived network contained 34,083 interactions between 9014 proteins. We determined edge reliability weights for these interactions with supporting evidence information including experimental validation, computational methods, and public literature mining for a number of proteins [45].

Transcriptional regulatory data was acquired from the Transfac Database (Release11.4) [46]. The resulting regulatory network consisted of 281 TFs and 624 genes with 1603 interactions. For further analysis, we assigned an empirical weight to be 0.99 (a balanced confidence level of each edge in a TRN) for each transcriptional regulatory interaction.

### Cancer mutated genes

Cancer mutated genes (384) were obtained from the Cancer Gene Census [47], a well-known online database cataloguing genes in which mutations have been causally implicated in a wide variety of tumour types.

### Human co-regulated module identification

#### Integrative weighted network construction

The human integrated network was represented as a weighted graph. The vertices of the graph were proteins or TFs, and the edges were protein-protein interactions or transcription regulation interactions. All edges are set confidence scores, as described above.

#### Probabilistic statistical model

We constructed a probabilistic model to evaluate whether a cluster of co-regulated proteins is likely to form a module in an integrated network. An underlying assumption was that a module corresponds to a sub-network that is typically dense. Under the probabilistic model, we formulated a log-likelihood ratio used to compare the fit of our model of a module against the likelihood that it arose at random. Highly scoring sub-networks corresponded to likely modules.

This approach requires constructing a sub-network model and a background model for interactions [48]. We defined two models: the sub-network model, *Ms*, assumed that interactions between proteins have a high probability *α *(set to 0.8), and that interactions between transcription factors and their target genes have high probability *β *(set to 0.9), according to the average level of the interaction's confidence weight in the real PPIN or TRN. In contrast, the background model, *Mb*, was obtained from a long series of random edge crosses by Monte Carlo simulations [49]. In this process, we chose two links *(a, b)*, *(c, d) *uniformly at random from the integrated network, and rewired them by exchanging their partners. Note that this procedure preserved their degrees distribution. We estimated the probabilities of interactions in the random network based on the percentage of the observed edges. We defined the likelihood model as:

Here, the ratio score of each candidate cluster is calculated by adding the log likelihood ratio score of the PPIN to that of the TRN. *P (u, v) *represents the confidence weight between two proteins *u *and *v*, and *P (u, t) *represents the confidence weight between protein *u *and transcription factor *t*. The probabilities *R (u, v) *and *R (u, t) *of the random network were estimated based on the percentage of the observed edge.

#### Module searching

Each candidate cluster was generated from our searching algorithm. The searching process consisted of three basic processes: *(i) *seed initialization; *(ii) *seed expanding; and *(iii) *overlap filtering.

We defined candidate seeds as a set with a TF and two of its binding genes, and restricted to include two protein-protein interactions. A greedy approach was used to filter the candidate seeds, retaining those with the highest L-score as the staring seed subunits.

In the second step, we expanded the starting seed subunits using a local search. In each seed expanding, we iteratively added a node (a protein or TF) to modify the current cluster, ensuring that each newly built candidate cluster had the highest ratio score. This procedure was repeated until the contribution gains passed a predefined threshold, which we defined as 4. Finally, after all expansion rounds, we checked overlaps between our resulting modules via a simple overlap ratio (*OR*):

Where *NO*_*i *_is the size of the overlaps between any two modules, and *NO*_*u *_is the union size of any two modules. If the *OR *score of two modules was larger than 0.8, we merge the module with lower L-score into larger one.

### Bridging co-regulated modules with biological entities using bipartite graphs

To access the packaging features of our resulting modules, we mapped them onto biological entities of genes, protein complexes, or metabolic pathways. For each module *M*, we constructed three 'Module-biological Entity' bipartite graphs: *(i) *G_M-g _= (M,g,E_M-g_) as a bipartite graph of module M-gene associations, where E_M-g _⊆ M × g; *(ii) *G_M-c _= (M,c,E_M-c_) as a bipartite graph of module M-complex associations, where E_M-c _⊆ M × c; and *(iii) *G_M-p _= (M,p,E_M-p_) as a bipartite graph of module M-pathway associations, where E_M-p _⊆ M × p;. Finally, we collected the biological relevance of our modules for rewiring different biological entities. As exemplified by cancer, we investigated whether cancer related co-regulated modules could assemble different cancer-related biological entities, and identified underlying biological associations of our co-regulated modules with cancer.

## Authors' contributions

LC, HW and LZ participated in the design of the study. WL, QW and YS carried out the integrated network construction. HW carried out co-regulated modules identification and analysis of the packaging feature. YH, WH, XL and JT participated in performance evaluation of the results. XL participated in the design and coordination of the study. All authors read and approved the final manuscript.

## Supplementary Material

Additional file 1 Information about the 96 co-regulated modules Click here for file

Additional file 2 Results of GO functional enrichment of co-regulated modules Click here for file

Additional file 3 Associations between co-regulated modules and cancer genes Click here for file

Additional file 4 Literature evidence for non-mutated genes in the 'biopolymer metabolic process' module Click here for file

Additional file 5 Complex packaging results for all co-regulated modules Click here for file

Additional file 6 Complex packaging results for 'biopolymer metabolic process' module Click here for file

Additional file 7 **Pathway packaging results for all co-regulated modules**. Click here for file
